# Tumor or Inflammatory Myofibroblastic Reaction in an Adolescent With an Abdominal Lymphatic Malformation?

**DOI:** 10.7759/cureus.23702

**Published:** 2022-03-31

**Authors:** Joseph J Lee, Romeo C Ignacio, Diego A Vicente, Angela M Bachmann, Pamela M Choi

**Affiliations:** 1 Department of Surgery, Naval Medical Center San Diego, San Diego, USA; 2 Division of Pediatric Surgery, Department of Surgery, University of California San Diego, Rady Children's Hospital, San Diego, USA; 3 Division of Surgical Oncology, Department of Surgery, Naval Medical Center San Diego, San Diego, USA; 4 Division of Gastrointestinal Pathology, Department of Pathology, Naval Medical Center San Diego, San Diego, USA; 5 Division of Pediatric Surgery, Department of Surgery, Naval Medical Center San Diego, San Diego, USA

**Keywords:** reactive myofibroblastic proliferation, abdominal lymphatic malformation, retrogastric mass, abdominal mass, abdominal lymphangioma

## Abstract

We report the case of a 17-year-old male who presented with intractable nausea and vomiting. Cross-sectional imaging revealed a large retrogastric abdominal mass. Fine needle aspiration done via endoscopic ultrasound (EUS) was nondiagnostic. Exploratory laparotomy revealed a large inflammatory mass densely adherent to the stomach and retroperitoneum. Incisional biopsy frozen section revealed spindle cells, and subsequent resection of the mass with en-bloc subtotal gastrectomy with Roux-en-y gastrojejunostomy reconstruction was performed. Final pathology demonstrated a lymphatic malformation with reactive myofibroblastic proliferation. Inflammatory abdominal lymphatic malformations are especially rare and not well described in the literature. These masses may present diagnostic challenges until the specimen is sent for pathologic analysis.

## Introduction

In the pediatric population, lymphatic malformations are rarely encountered in visceral locations, and the abdominal cavity comprises less than 5% of all lymphatic malformations [1]. Reactive myofibroblastic proliferation is particularly not well described in the literature. This case emphasizes the need for high clinical suspicion for this rare diagnosis to avoid misdiagnosis and receive the correct treatment.

## Case presentation

A previously healthy 17-year-old male presented with a four-day history of nausea, vomiting, and mild periumbilical abdominal discomfort. He had no constitutional symptoms and no other significant medical or surgical history. He was hemodynamically stable and afebrile. The physical exam was notable for a BMI of 30.8 with a nontender abdominal exam. The labs were unremarkable. A CT scan demonstrated a large, irregular, and multiloculated abdominal mass adjacent to the stomach and pancreas, measuring up to 8.3 cm at its largest diameter.

The patient was sent for endoscopic ultrasound (EUS) with biopsy. The EUS identified a multiloculated cystic mass distinct from the stomach. A fine-needle aspiration (FNA) was performed and demonstrated scant fragments of fibrovascular tissue, unremarkable gastrointestinal epithelium, and small mature lymphocytes.

**Figure 1:**
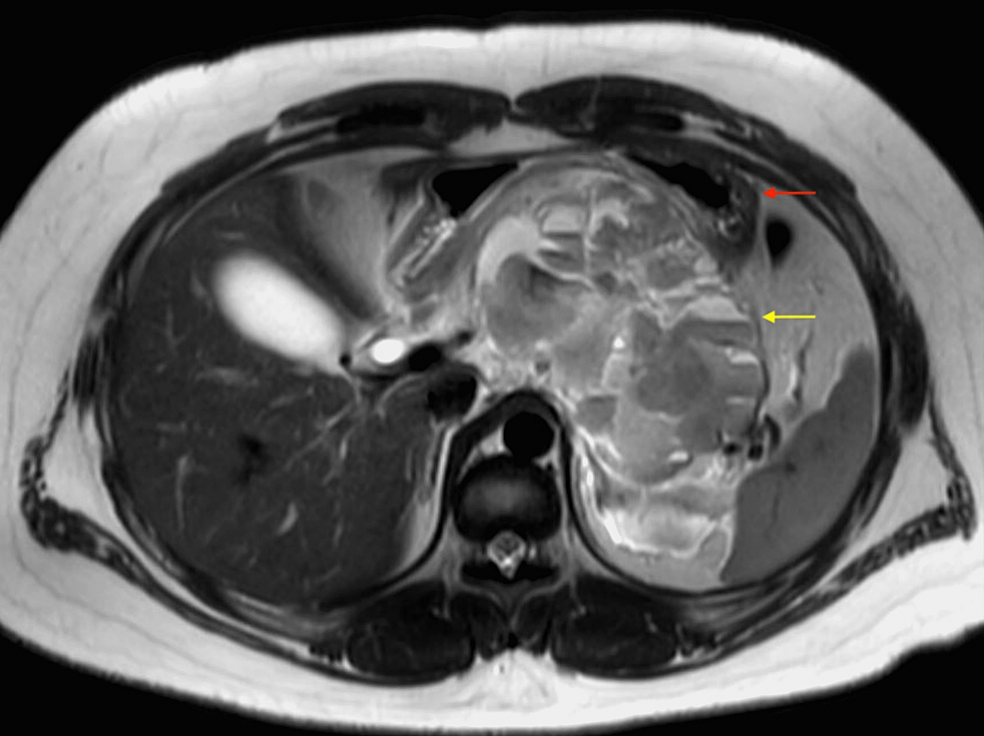
MRI of abdomen with lobulated intra-abdominal mass (yellow arrow) posterior to stomach (red arrow).

A subsequent abdominal MRI identified a lobulated mass within the lesser sac of the peritoneal cavity, measuring 10.9 cm × 6.7 cm × 6.1 cm, with multiple internal fluid-fluid levels (Figure 1). The lesser gastric artery and its distal branches were interspersed within this mass. The mass was abutting the lesser curvature of the stomach. However, there were no radiological findings to suggest an invasion of the stomach wall. Imaging features of the mass were consistent with possible lymphatic malformation or sarcoma.

**Figure 2:**
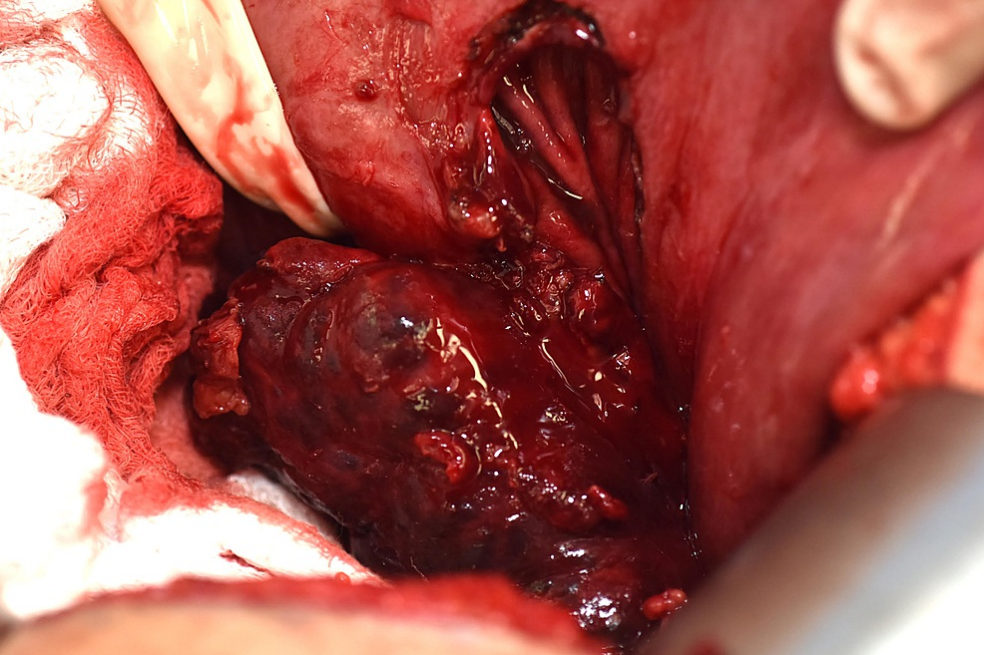
Intraoperative image of mass separated from stomach involving gastric tissue.

Given that imaging and FNA were unable to establish a definitive diagnosis, resection of the mass for symptom relief and tissue diagnosis was indicated. We proceeded with an exploratory laparotomy with a plan for excision of the mass. Intra-operatively, the lesser sac space was entered, and the mass was encountered. However, it was quite firm and densely adherent to the retroperitoneum as well as the posterior wall of the stomach along the lesser curvature (Figure 2). The mass was dissected away from the pancreas and attempts to separate the mass from the stomach wall resulted in bleeding and violation of the stomach wall.

**Figure 3:**
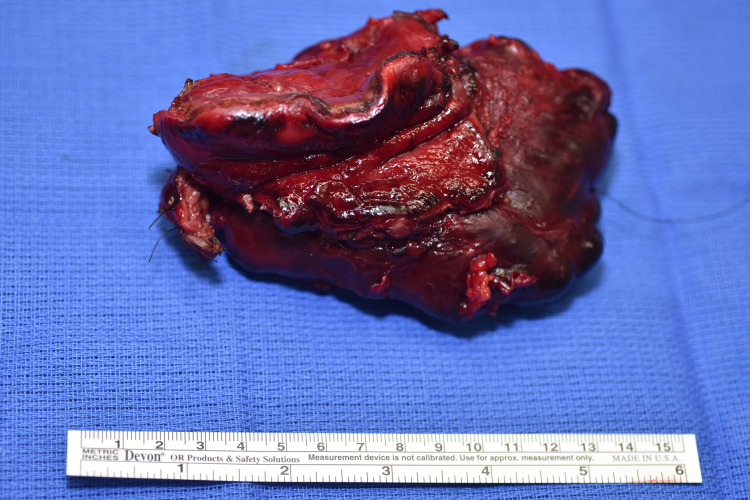
Gross surgical specimen of multilobulated lymphatic malformation involving gastric tissue.

**Figure 4:**
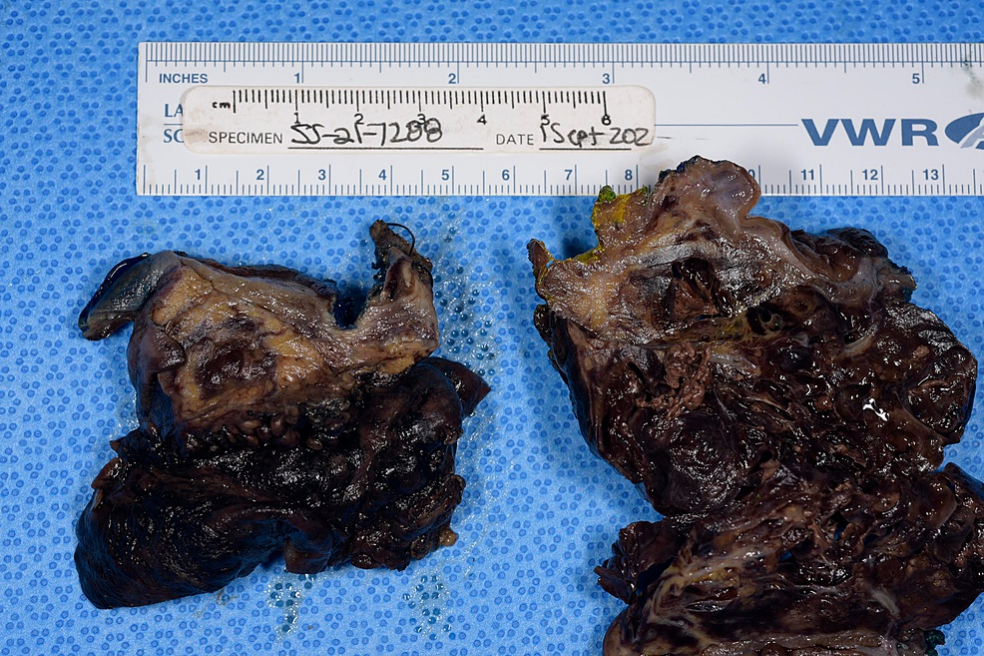
Cut section of the mass revealing dilated cysts.

An incisional biopsy of the mass was sent for frozen section pathology, which revealed spindle cells without clear evidence of benign versus malignant features. Given the broad differential as well as concern for malignancy, the decision was made to perform a subtotal gastrectomy in order to completely resect the mass along with retro-colic gastrojejunostomy Roux-en-Y reconstruction (Figures 3 and 4).

**Figure 5:**
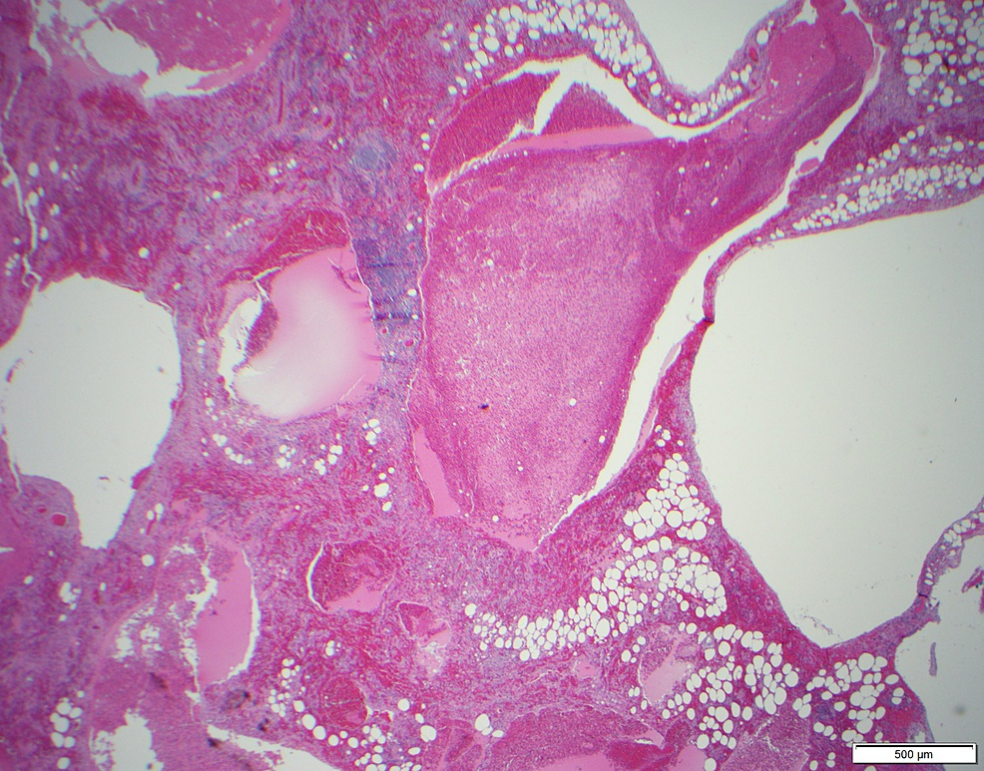
Low power view (40×) demonstrates a mass-like lesion in the perigastric soft tissue with multi-loculated cystic spaces containing blood and proteinaceous material.

**Figure 6:**
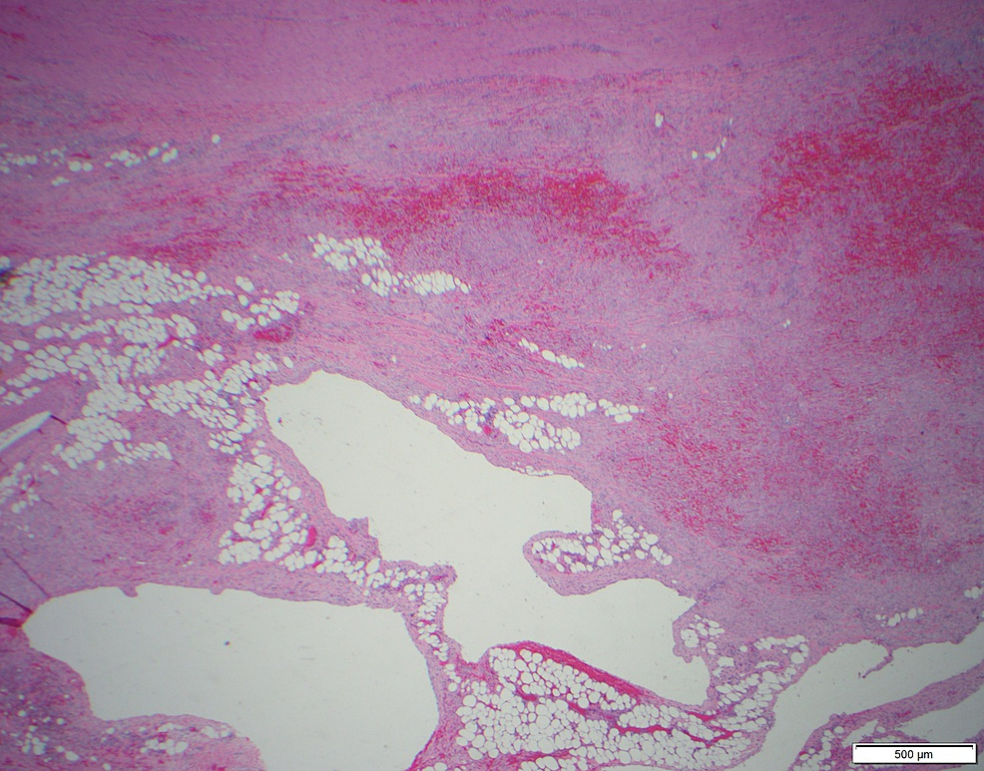
Low power view (40×) with gastric wall along the top border of the picture.

**Figure 7:**
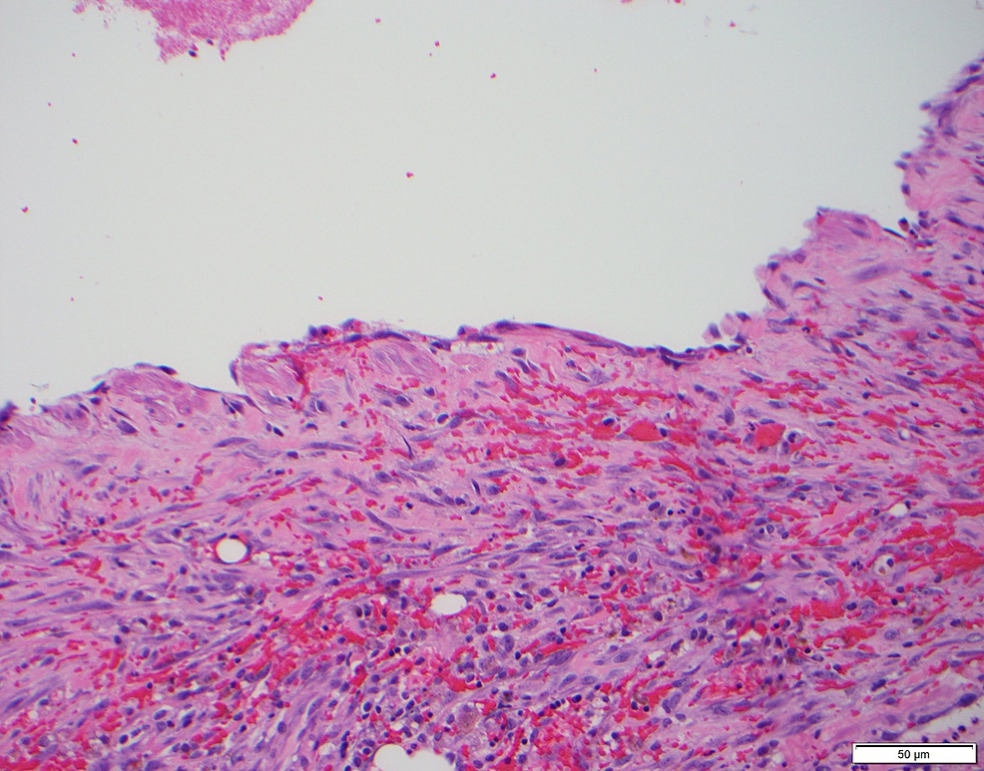
The cystic spaces are lined by bland-appearing flat endothelial cells. Hemorrhage and a myofibroblastic proliferation are seen between the cystic spaces (200×).

**Figure 8:**
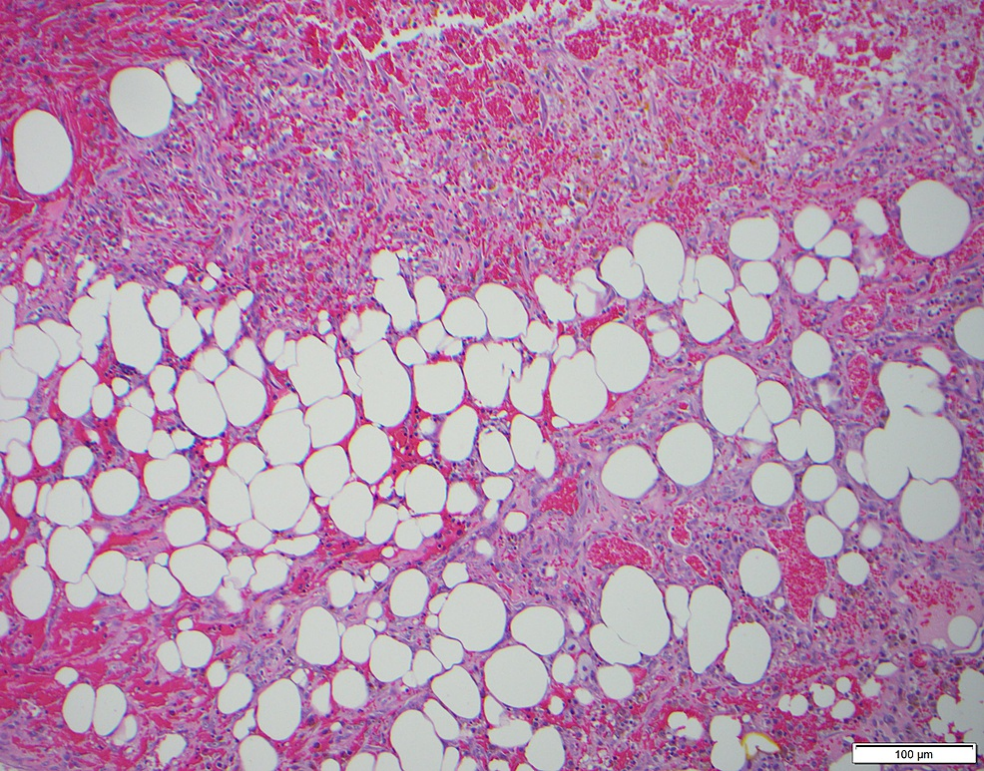
Much of the lesion contains extensive hemorrhage with hemosiderin deposition and is composed of a myofibroblastic proliferation with reactive cytologic features and associated inflammatory cells (100×).

**Figure 9:**
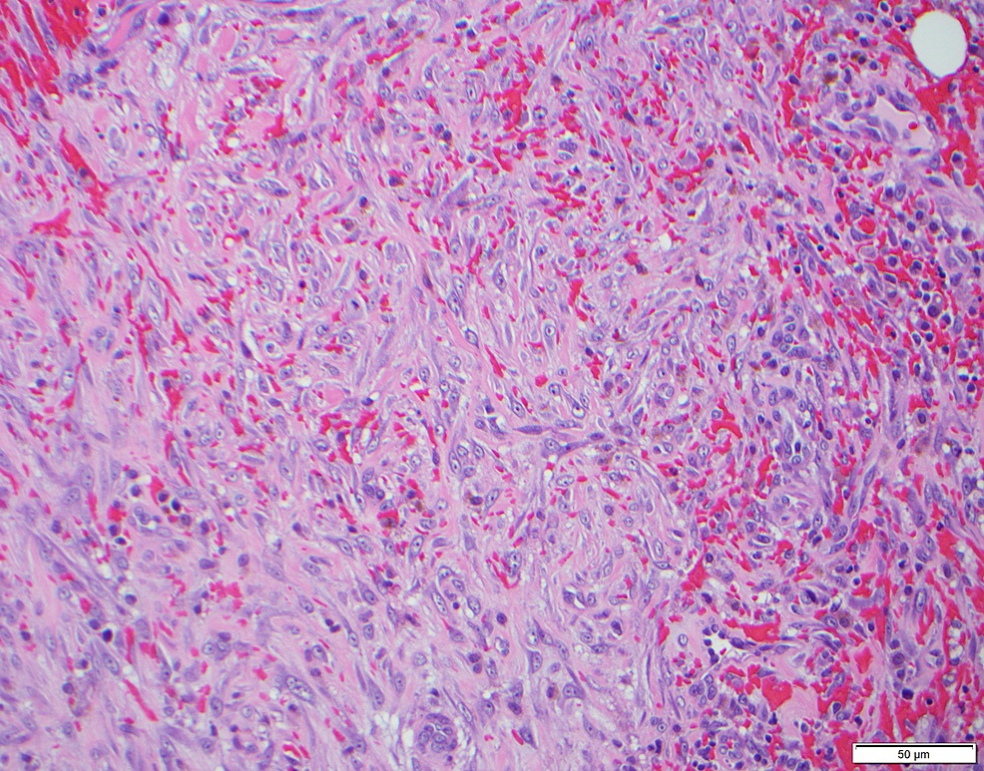
Extensive hemorrhage with hemosiderin deposition composed of a myofibroblastic proliferation with reactive cytologic features and associated inflammatory cells under higher magnification (200×).

The surgical specimen was sent to the Mayo Clinic for specialty staining. The final pathology diagnosis was lymphatic malformation with extensive hemorrhage and reactive myofibroblastic proliferation in the perigastric soft tissue (Figures 5-9). At six months post-surgery, a surveillance MRI demonstrated no signs of recurrence.

## Discussion

Lymphatic malformations are congenital malformations defined by the proliferation of dilated lymphatic vessels that are lined by endothelial cells during the embryonic period. As lymphatic channels are found throughout the entire body, lymphatic malformations may be present in many anatomic locations, but most commonly in the head and neck (70-75%) [2].

Abdominal lymphatic malformations represent less than 5% of all lymphatic malformations [1,3,4]. Within the abdomen, lymphatic malformations are found in various locations, including the mesentery, retroperitoneum, omentum, pancreas, and spleen. They have a wide range of clinical presentations, ranging from asymptomatic to abdominal pain, nausea/vomiting, and distention [1,3-9]. Significant complications of these abdominal lymphatic malformations include obstruction, infection, torsion/volvulus, hemorrhage, rupture, and peritonitis [5,10-12]. Pediatric patients with abdominal lymphatic malformations tend to present with more acute symptoms as opposed to adults, who tend to be either asymptomatic or present with chronic symptoms [3].

Some studies have demonstrated the progression of lymphatic malformations, defined as enlargement or the onset/worsening of symptoms. One study found that children had a 42.2% risk of progression before adolescence, 84.7% before adulthood, and 95.3% during their lifetime. Adolescents were particularly at the greatest risk for progression, so this process is hypothesized to be hormone modulated [13]. While high rates of regression have been noted in asymptomatic head and neck lymphatic malformations [14], the true progression or regression rate in abdominal lymphatic malformations is unknown due to their overall rarity. Many tend to be symptomatic at presentation and so are treated at the time of diagnosis.

Diagnosing abdominal lymphatic malformations begins with radiological studies to identify and characterize the tumor. While some may be diagnosed by ultrasound as a first-line study, MRI is often required to better characterize the mass, particularly with regard to cyst size (macrocystic or microcystic), loculations versus septations, focal versus diffuse, as well as its relationship to surrounding structures [15,16]. Despite advanced imaging, preoperative diagnosis may still be challenging until pathology can be obtained.

The management of these abdominal lymphatic malformations continues to change and evolve. While surgical excision has been the mainstay of treatment and should still be considered in symptomatic patients, medical treatment with sirolimus and sclerotherapy injections has also been demonstrated to be efficacious, particularly as these masses may be intimately associated with critical structures [17,18]. Imaged-guided sclerotherapy has been found to be efficacious for macrocystic lymphatic malformations, particularly with repeat treatments [18,19]. Microcystic lymphatic malformations may be treated with sirolimus, an inhibitor of the mammalian target of rapamycin complex 1, which has also resulted in significant regression [18]. However, neither sclerotherapy nor sirolimus was considered viable treatment options for our patient, as the diagnosis was not confirmed based on imaging, and the patient was symptomatic. 

The occurrence of inflammatory changes in an abdominal lymphatic malformation is rarely described in the literature, especially in the pediatric population. A small case series identified seven inflammatory abdominal lymphatic malformations, of which five were in adults. The two pediatric cases were at the ages of one month and five years. All seven patients were symptomatic. The locations were also varied, with three cases in the mesentery of the small intestine and four cases in the retroperitoneum. On gross examination, the tumors were multiloculated and cystic masses with areas of fat necrosis and hemorrhage. Histologically, all seven cases were marked by extensive areas of granulation tissue, both chronic and occasional foci of acute inflammation, with a floridly cellular reactive myofibroblastic proliferation. These findings describe the tendency of lymphatic malformations to induce marked reactive inflammatory changes in the surrounding tissues [20]. Additionally, we identified one other case in the literature in which a 65-year-old man had a very similar course to our patient. He presented with gastric outlet obstruction, and imaging studies found a posterior antral mass, but FNA identified spindle cells. An oncologic gastric resection was performed, and pathology also revealed lymphatic malformation with fibroblastic and myofibroblastic proliferation [10].

Given that this inflammatory reaction often obscures the benign nature of these lymphatic masses, occasionally leading to the clinical impression of a malignant tumor, awareness of the rare occurrence is required to help correct the diagnosis.

## Conclusions

Abdominal lymphatic malformations are rare in the pediatric population and require a high level of clinical suspicion to avoid misdiagnosis and to receive the correct treatment. Reactive inflammatory lymphatic malformations are a particularly rare variant of lymphatic malformations, and given the propensity to involve adjacent structures, preoperative considerations of this diagnosis may aid in operative planning.

Our literature review reveals that while surgical excision has been the mainstay of treatment and should still be considered in symptomatic patients, medical treatment with sirolimus and sclerotherapy injections has also been demonstrated to be efficacious in many patients. However, sirolimus and sclerotherapy were not considered viable options for our patient as the diagnosis was not established based on advanced imaging. This rare inflammatory reaction in lymphatic malformations often obscures the benign nature of these lymphatic masses, occasionally leading to the clinical impression of a malignant tumor. Clinicians should be aware of the possibility of this entity when considering the differential diagnosis of uncommon abdominal masses.

## References

[1] Mahle C, Schwartz M, Popek E, Bocklage T (1997). Intra-abdominal lymphangiomas in children and adults. Assessment of proliferative activity. Arch Pathol Lab Med.

[2] Schoinohoriti OK, Theologie-Lygidakis N, Tzerbos F, Iatrou I (2012). Lymphatic malformations in children and adolescents. J Craniofac Surg.

[3] Goh BK, Tan YM, Ong HS (2005). Intra-abdominal and retroperitoneal lymphangiomas in pediatric and adult patients. World J Surg.

[4] Kosir MA, Sonnino RE, Gauderer MW (1991). Pediatric abdominal lymphangiomas: a plea for early recognition. J Pediatr Surg.

[5] Alfadhel SF, Alghamdi AA, Alzahrani SA (2019). Ileal volvulus secondary to cystic lymphangioma: a rare case report with a literature review. Avicenna J Med.

[6] Steyaert H, Guitard J, Moscovici J, Juricic M, Vaysse P, Juskiewenski S (1996). Abdominal cystic lymphangioma in children: benign lesions that can have a proliferative course. J Pediatr Surg.

[7] Kopicky L, Humenansky KM, Gitzelmann C, Gulati R (2017). Intraabdominal cystic lymphangioma. J Ped Surg Case Rep.

[8] Kenney B, Smith B, Bensoussan AL (1996). Laparoscopic excision of a cystic lymphangioma. J Laparoendosc Surg.

[9] Méndez-Gallart R, Bautista A, Estévez E, Rodríguez-Barca P (2011). Abdominal cystic lymphangiomas in pediatrics: surgical approach and outcomes. Acta Chir Belg.

[10] Nayak M, Purkait S, Sasmal PK, Singh PK (2020). Cystic lymphangioma of the stomach with marked reactive changes: a rare cause of gastric outlet obstruction in adult. BMJ Case Rep.

[11] Gasparella P, Singer G, Castellani C, Sorantin E, Haxhija EQ, Till H (2020). Giant lymphatic malformation causing abdominal compartment syndrome in a neonate: a rare surgical emergency. J Surg Case Rep.

[12] Méndez-Gallart R, Solar-Boga A, Gómez-Tellado M, Somoza-Argibay I (2009). Giant mesenteric cystic lymphangioma in an infant presenting with acute bowel obstruction. Can J Surg.

[13] Hassanein AH, Mulliken JB, Fishman SJ, Quatrano NA, Zurakowski D, Greene AK (2012). Lymphatic malformation: risk of progression during childhood and adolescence. J Craniofac Surg.

[14] Thorburn C, Price D (2022). Expectant management of pediatric lymphatic malformations: a 30-year chart review. J Pediatr Surg.

[15] Hinds DF, Aponte EM, Secko M, Mehta N (2015). Identification of a pediatric intra-abdominal cystic lymphangioma using point-of-care ultrasonography. Pediatr Emerg Care.

[16] Francavilla ML, White CL, Oliveri B, Lee EY, Restrepo R (2017). Intraabdominal lymphatic malformations: Pearls and pitfalls of diagnosis and differential diagnoses in pediatric patients. AJR Am J Roentgenol.

[17] Kulungowski AM, Patel M (2020). Lymphatic malformations. Semin Pediatr Surg.

[18] Zamora AK, Barry WE, Nowicki D (2021). A multidisciplinary approach to management of abdominal lymphatic malformations. J Pediatr Surg.

[19] Madsen HJ, Annam A, Harned R, Nakano TA, Larroque LO, Kulungowski AM (2019). Symptom resolution and volumetric reduction of abdominal lymphatic malformations with sclerotherapy. J Surg Res.

[20] Hornick JL, Fletcher CD (2005). Intraabdominal cystic lymphangiomas obscured by marked superimposed reactive changes: clinicopathological analysis of a series. Hum Pathol.

